# Dabigatran Versus Warfarin After Bioprosthesis Valve Replacement for the Management of Atrial Fibrillation Postoperatively: Protocol

**DOI:** 10.2196/resprot.3014

**Published:** 2014-04-01

**Authors:** Andre Rodrigues Duraes, Pollianna DS Roriz, Fabio V Bulhoes, Bianca DA Nunes, Juliana QV Muniz, Italvar NDCR Neto, Andre MS Fernandes, Francisco JFBD Reis, Edmundo JN Camara, Erenaldo DSR Junior, Deusdeth TS Segundo, Felipe Pinho E Albuquerque Silva, Roque Aras

**Affiliations:** ^1^Hospital Ana NeryServiço de CardiologiaUniversidade do Estado da BahiaSalvadorBrazil; ^2^Hospital Ana NeryServiço de CardiologiaUniversidade Federal da BahiaSalvadorBrazil

**Keywords:** warfarin, dabigatran, anticoagulants, atrial fibrillation, stroke, hematologic agents

## Abstract

**Background:**

Warfarin and similar vitamin K antagonists have been the standard therapy for patients with mechanical or biological valve prosthesis and atrial fibrillation (AF). Even with the appropriate use of therapy, some studies have reported that there is a high incidence of thromboembolic events, 1%-4% per year. Furthermore, a bleeding risk is significant, ranging from 2% to 9% per year, according to some studies.

**Objective:**

The objective of our study was to examine the effect of dabigatran etexilate versus dose-adjusted warfarin for the prevention of intracardiac thrombus in persistent or permanent AF at least 3 months after aortic and/or mitral bioprosthesis replacement.

**Methods:**

Dabigatran versus warfarin after bioprosthesis valve replacement for the management of atrial fibrillation postoperatively (DAWA) is a phase 2, prospective, open label, randomized exploratory pilot study. The main variable to be observed in this study is intracardiac thrombus. From August 2013 to April 2015, 100 patients, at least 3 months after aortic and/or mitral bioprosthesis replacement and permanent or persistent AF postoperatively, who match eligibility criteria will be selected from Ana Nery Hospital in Salvador-Bahia with a follow-up of three months. Patients were randomly assigned in a 1:1 ratio to receive either dabigatran etexilate or warfarin.

**Results:**

Although the present study has no statistic power to proof non-inferiority, it is expected that the dabigatran etexilate group will be protected as well as the warfarin group from intracardiac thrombus, without increasing the bleeding rates, since we are using safer doses (110 mg bid). The lack of necessity of monitoring INR is also another factor that contributes to a better adherence to the new drug and it can make all the difference in the manner of doing anticoagulation for patients with similar clinical characteristics.

**Conclusions:**

The study is in the recruitment phase. It is possible that dabigatran etexilate is as effective as warfarin in preventing the emergence of intracardiac thrombus in patients with AF and mitral and/or aortic bioprosthesis.

**Trial Registration:**

Clinicaltrials.gov NCT01868243; http://clinicaltrials.gov/ct2/show/NCT01868243 (Archived by WebCite at http://www.webcitation/6OABiuasd).

## Introduction

### Overview

Valvular heart disease affects over 100 million people worldwide. Its prevalence is rising due to the high incidence of rheumatic disease in developing countries and the growing impact of degenerative valve disease in elderly populations, a consequence of population ageing [1,2]. An estimated 4 million valve replacement procedures have been performed in the last 50 years, and it remains the only definitive treatment for most patients with advanced heart valve disease [3].

### Warfarin

Warfarin and similar vitamin K antagonists (VKA) have been the standard therapy for patients with a prosthetic heart valve and atrial fibrillation (AF). Even with the appropriate use of therapy, the incidence of thromboembolic events is still substantial, 1%-4% per year. Furthermore, bleeding risk is significant, ranging from 2% to 9% per year [3-5]. VKA have a narrow therapeutic index and also a complex pharmacology, for example, a long pharmacologic inertia and common interaction with other drugs [6]. These features make the management of these drugs a challenge for physicians and their patients.

### Dabigatran Etexilate

Dabigatran etexilate is the prodrug of dabigatran, a direct thrombin (factor IIa) inhibitor. After administration, it is rapidly converted by serum esterase to the active moiety, dabigatran, which is a nonpeptide, potent, competitive, and reversible inhibitor of thrombin. The pharmacokinetics and pharmacodynamics of dabigatran allow fixed dose administration without coagulation monitoring [7].

The US Food and Drug Administration (FDA) approved dabigatran on October 19, 2010, for the prevention of stroke and systemic embolism in patients with nonvalvular AF [8]. The Randomized Evaluation of Long-Term Anticoagulation Therapy (RE-LY) trial [9] was a noninferiority study that compared dabigatran at 2 ﬁxed doses (150 mg twice daily and 110 mg twice daily) with dose-adjusted warfarin (target international normalized ratio, INR range = 2.0-3.0) reaching the criterion of noninferiority for the two doses tested (relative risk, RR = 0.66; 95% conﬁdence interval, CI 0.53-0.82; *P*=.001; and RR=0.91; 95% CI 0.74-1.11; *P*=.34, respectively) [8]. The continued observation of 5851 dabigatran-treated RE-LY patients beyond the randomized trial's follow-up time suggests no significant difference between the two dosages in the trial's primary endpoint, stroke or systemic embolism, however, the 150 mg dosage showed higher rates of major and minor bleeding, without increasing the risk of hemorrhagic stroke [10].

Nevertheless, there are no studies in humans evaluating the efficacy and safety of dabigatran in patients with AF and mitral and/or aortic bioprosthetic valve. Among patients with mechanical heart valves, the randomized phase II study to evaluate the safety and pharmacokinetics of oral dabigatran etexilate in patients after heart valve replacement (RE-ALIGN) was terminated early because the dabigatran etexilate treatment arm had significantly more thromboembolic events (valve thrombosis, stroke, and myocardial infarction) and major bleeding (predominantly postoperative pericardial effusions requiring intervention for hemodynamic compromise) than did the warfarin treatment arm. These bleeding and thromboembolic events were reported in patients who were initiated on dabigatran etexilate postoperatively within 3 days after mechanical bileaflet valve implantation, and in patients whose valves had been implanted more than 3 months previously [11].

With this gap in mind, considering the complicated management of VKAs, the investigators of this study chose to conduct an open randomized controlled pilot trial to assess the efficacy and safety of dabigatran etexilate compared with warfarin in patients with persistent or permanent AF after bioprosthetic mitral and/or aortic replacement (DAWA).

## Methods

### Study Design

DAWA is a phase 2, prospective, open label randomized pilot study. The main variable to be observed in this study is intracardiac thrombus. There are no formal primary or secondary clinical efﬁcacy or safety outcomes because it is a pilot study. Mortality and morbidity events (ischemic and hemorrhagic stroke, systemic embolism, major bleeding, prosthesis valve thrombosis, and any cause of death) will be evaluated in an exploratory manner and are considered as further endpoints in this trial. The inclusion and exclusion criteria are outlined in Textboxes 1 and 2.

The study will be performed in accordance with the ethical principles that have been laid down in the Declaration of Helsinki. They are consistent with the International Conference on Harmonization/Good Clinical Practice, and applicable regulatory requirements in Brazil where it was approved unreservedly by the local ethics and research committee´s under protocol number 14284813.9.0000.0045. A data monitoring committee will monitor patient safety and treatment efficacy every 2 months.

**DAWA inclusion criteria.**

1. Age from 18 to 64 years at entry
2. Patients with mitral and/or aorthic valve bioprosthesis for at least 3 months postoperatively
3. There is 12-lead electrocardiogram documented AF on the day of screening or randomization; or a 24-hour Holter electrocardiogram recording showing AF episodes postoperatively
4. Brain computed tomography scan without hemorrhage or findings of acute cerebral infarction on the last 2 days of screening
5. Exclusion of atrial thrombus or valve prosthesis thrombosis by transesophageal echocardiograph on the last 2 days of screening
6. Written, informed consent

**DAWA exclusion criteria.**

1. Previous hemorrhagic stroke
2. Ischemic stroke in the last 6 months
3. Severe renal impairment (creatinine clearance rates < 30 ml/min)
4. Active liver disease (any etiology)
5. Concomitant use of any antiplatelet (aspirin, clopidogrel, prasugrel, ticagrelor, ticlopidine, etc)
6. Increased risk of bleeding (congenital or acquired)
7. Uncontrolled hypertension
8. Gastrointestinal hemorrhage within the past year
9. Anemia (hemoglobin level <10 g/dL) or thrombocytopenia (platelet count < 100 × 109/L)
10. Active infective endocarditis
11. Pregnant or lactating women

### Randomization and Follow-Up

Prospectively, from August 2013 to April 2015, 100 consecutive patients between 18 and 64 years old, at least 3 months after mitral and/or aortic bioprosthesis valve replacement, documented AF postoperatively, and who match the eligibility criteria are being recruited from Ana Nery Hospital in Salvador-Brazil after obtention of informed consent.

**Figure 1:**
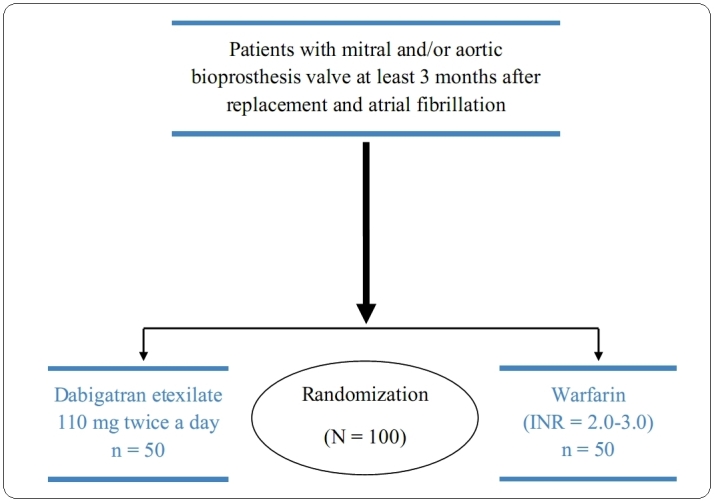
DAWA study.

For the allocation of the participants, a computer generated list of random numbers including 1 to 100 was used [12]. Following that, the allocation sequence was concealed (in sequentially numbered, opaque, black, sealed, and stapled envelopes) from the researcher who was enrolling and assessing participants. By convention, every “even number” is being allocated to the dabigatran etexilate group and every “odd number” to the warfarin group, that was consecutively selected blindly from the previous list, thereby selecting exactly 50 patients in each group (1:1 allocation), (Figure 1 shows this grouping). After this randomization, patients had study visits scheduled at 7 days (via telephone) and at 30 days (personally). The enrollment period was 21 months with a planned minimum follow-up period of 3 months.

### Drug Administration Protocol

Dabigatran etexilate will be supplied as capsules containing 110 mg, which should be taken twice daily without the need of INR monitoring. All the patients randomized to use dabigatran etexilate will remain with that drug for the duration of the study. Any patients who used warfarin previously will do a drug washout, followed by an immediate introduction of dabigatran etexilate once the INR < 2.5.

The patients assigned to warfarin will require close coagulation monitoring to achieve the target INR (range, 2.0-3.0). Therefore, it will be necessary to follow a protocol of monitoring and dosage adjustment of this drug to ensure the safety and optimization of its use since each patient responds differently to the same doses. The warfarin dose adjustment algorithm was made according to the evidence-based guideline of the American College of Chest Physicians, and several other studies [6,13-18]. Doses between 5 mg and 10 mg were administered for the first days for most individuals, with subsequent dosing based on INR response. The patients should have a baseline prothrombin time/INR checked prior to initiating warfarin therapy, and rechecked 2-3 days after initiating therapy, and then checked again every 2-3 days until stable. After this, they will be rechecked after 15 days, and if they are stable, every 30 days.

### Outcomes

The primary endpoint will be the appearance of an intracardiac thrombus in an esophageal echocardiogram to be held at the end of the follow-up. Other probable endpoints are the composite of stroke (ischemic or hemorrhagic), systemic embolism (to the central nervous system or peripheral), and prosthesis valve thrombosis. The safety endpoints will be any bleeding event (major or minor), elevated liver enzymes, or hepatic function abnormalities.

A thromboembolic event involving the central nervous system was defined as a sudden, focal neurological deficit of presumed vascular origin lasting 24 hours to 7 days (reversible ischemic neurological deficit) or enduring more than 7 days (stroke), as confirmed by computed tomographic or magnetic resonance scan imaging and evaluated by a skilled physician. A peripheral embolism was diagnosed when there was a sudden onset of arterial occlusion in the extremities (with or without cyanosis) and with reported absence of pulse, or sudden abdominal pain requiring urgent intervention (confirming acute intestinal ischemia during the surgical procedure). One of the criteria in the list below defined a major bleeding event [19]. All other bleeding will be considered minor and will also be computed.

Major bleeding criteria: (1) fatal bleeding, (2) symptomatic bleeding in a critical area or organ, such as intracranial, intraspinal, intraocular, retroperitoneal, intraarticular or pericardial, or intramuscular with compartment syndrome, and/or, (3) bleeding causing a fall in hemoglobin level of 20 g L^-1^(1.24 mmol L^-1^) or more, or leading to transfusion of two or more units of whole blood or red cells.

### Safety Monitoring

Any patients with bleeding on dabigatran etexilate or warfarin therapy will be immediately hospitalized and screened for the severity of bleeding (mild, moderate, or life-threatening bleeding). The management of these complications will follow current guidelines and review articles [13-18].

All participants will have telephone visits scheduled weekly. After that period, study visits will be scheduled monthly. The telephone number of all the researchers will also be available to the patients. At any time, in any phase of the study, the patient will have the full right to request exclusion from the study.

*Electrocardiography* and laboratory tests (blood count, renal function, electrolytes, and liver function tests) will be performed monthly to monitor any changes that indicate bleeding or coagulopathy. Before the randomization and after the follow-up period, patients will undergo a brain computed tomography without contrast to exclude recent ischemic or previous hemorrhagic cerebrals events, and a transesophageal echocardiogram to check prosthesis function (including peak and mean gradient), and to check left atrial thrombus.

During the follow-up, if any unfavorable outcome with patients in the study is evidenced, an emergency meeting with the ethics committee will be held to evaluate the discontinuation of the study, regardless of their stage of recruitment. Other than that, every 2 months there will be a meeting with the same group with the intention of observing the progress of the study and the outcomes observed in each patient.

### Statistical Considerations

The SPSS 17.0 (SPSS Inc) will be used to perform statistical analysis of the collected data. The primary analysis will be performed according to the intent-to-treat principle. A safety analysis will be performed on all patients treated regardless of any protocol violations. The quantitative variables will be described as mean and standard deviation. The mean comparison will be performed by the Student *t* test for independent populations or related populations, as appropriate. The qualitative and categorical variables will be presented as percentages and their comparisons will be made by χ2 test (chi-square) or the Fisher exact test when indicated.

### Use of Concomitant Drugs

The use of drugs such as acetylsalicylic acid, clopidogrel, and other antiplatelet agents will not be allowed during the study period. The same applies to antiarrhythmic drugs that interact with dabigatran etexilate, such as quinidine.

## Results

Although the present study has no statistic power to proof non-inferiority, it is expected that the dabigatran etexilate group will be protected as well as the warfarin group from intracardiac thrombus, without increasing the bleeding rates, since we are using safer doses (110 mg bid). The lack of necessity of monitoring INR is also another factor that contributes to a better adherence to the new drug and it can make all the difference in the manner of doing anticoagulation for patients with similar clinical characteristics. Enrollment for this study started in July 2013 and is expected to conclude in April 2015. Final results are expected in September 2015. The study is in the recruitment phase.

## Discussion

Anticoagulant treatment reduces the incidence of death and cardioembolic events in patients with AF or a prosthetic heart valve, and the incidence of death and recurrences in patients with venous thromboembolism [18]. Warfarin works by binding to vitamin K epoxide reductase to inhibit vitamin K-dependent coagulation factors II, VII, IX, and X. For all its extensive use, warfarin has many clinical shortcomings, including variable pharmacokinetic and pharmacodynamics properties, a narrow therapeutic index range, and numerous interactions with certain foods and drugs. All of these factors contribute to the need for frequent coagulation laboratory monitoring and dosage adjustments.

Recently, direct thrombin inhibitors and factor Xa inhibitors have been added to the armamentarium for anticoagulation in AF. Dabigatran is an oral, reversible, direct competitive inhibitor of thrombin, which prevents the conversion of ﬁbrinogen to ﬁbrin within the coagulation cascade, thereby inhibiting thrombus formation [8]. The association of AF and bioprosthesis increases significantly the thromboembolic risk, making the use of dabigatran etexilate uncertain at this scenario.  The RE-ALIGN trial [20] was the first clinical study to evaluate an oral direct thrombin inhibitor (dabigatran etexilate) as an alternative to warfarin for use in patients with mechanical heart valves requiring anticoagulation therapy. That study was prematurely interrupted because it showed increased rates of thromboembolic and bleeding events in the dabigatran etexilate treatment arm [20]. This report describes the rationale and design of the first clinical trial, to our knowledge, to test the hypothesis that dabigatran etexilate exhibits similar efficacy to warfarin with respect to prevention of intracardiac thrombus in patients with aortic and/or mitral bioprosthesis valve and AF postoperatively.

## Abbreviations

AF: atrial fibrillation
CI: conﬁdence interval
DAWA: dabigatran compared with warfarin in patients with persistent or permanent AF after bioprosthetic mitral and/or aortic replacement
FDA: US Food and Drug Administration
INR: international normalized ratio
RE-ALIGN: randomized, phase II study to evaluate the safety and pharmacokinetics of oral dabigatran etexilate in patients after heart valve replacement
RE-LY: randomized evaluation of long-term anticoagulation therapy
RR: relative risk
VKA: vitamin K antagonists
